# Increased cancer stem cell invasion is mediated by myosin IIB and nuclear translocation

**DOI:** 10.18632/oncotarget.9896

**Published:** 2016-06-07

**Authors:** Dustin Thomas, Praveena S. Thiagarajan, Vandana Rai, Ofer Reizes, Justin Lathia, Thomas Egelhoff

**Affiliations:** ^1^ Department of Cellular and Molecular Medicine, Lerner Research Institute, Cleveland Clinic, Cleveland, OH, USA; ^2^ Department of Molecular Medicine, Cleveland Clinic Lerner College of Medicine of Case Western Reserve University, Cleveland, OH, USA

**Keywords:** cancer stem cell, myosin IIB, nuclear translocation, invasion, breast cancer

## Abstract

Despite many advances in the treatment of breast cancer, it remains one of the leading causes of death among women. One hurdle for effective therapy is the treatment of the highly invasive and tumorigenic subpopulation of tumors called cancer stem cells (CSCs). CSCs, when stimulated with EGF, migrate through a physiological 3D collagen matrix at a higher velocity than non-stem cancer cells (non-SCCs). This increased invasion is due, in part, by an enhanced nuclear translocation ability of CSCs. We observed no difference between CSC and non-SCC in cellular migration rates on a 2D surface. Furthermore, during transwell migration using large diameter transwell pores, both CSC and non-SCC populations migrated with similar efficiency. However, when challenged with more restrictive transwells, CSCs were dramatically more capable of transwell migration. These results implicate nuclear translocation as a major rate limiting factor for CSC dissemination. We further show that non-muscle myosin IIB is critical for this enhanced nuclear translocation and the ability for cancer stem cells to efficiently migrate through restrictive 3D environments. These studies suggest that cytoskeletal elements upregulated in CSCs, such as myosin IIB, may be valuable targets for intervention in cancer stem cell dispersal from tumors.

## INTRODUCTION

Breast cancer is one of the leading causes of death among women in the United States with an estimated 300,000 new diagnoses in 2013 [1]. Within solid tumors such as breast [2], brain [3], prostate [4] and lung [5] cancer, as well as hematological malignancies such as multiple myeloma [6], a sub population of cells exist that contain properties for self-renewal and the ability to initiate tumor formation, commonly referred to as cancer stem cells (CSCs). These stem cell behaviors are driven by the expression of master regulator transcription factors, such as OCT4, NANOG and SOX2 [7]. CSCs present themselves as difficult barriers in the treatment of cancer for a number of reasons. First, CSCs have an enhanced survivability compared to non-stem cancer cells (non-SCCs). Not only are they able to outcompete non-SCCs for available resources [8], CSCs are more resistant to current therapeutics [9]. This drug resistance of tumor initiating CSCs is believed to be a key cause of the development of drug resistance of tumor recurrence in patients [10, 11]. The second aspect of CSCs that contributes to their progression of cancer is their ability to invade and metastasize at secondary sites [12]. A widely studied area of cancer biology is the epithelial to mesenchymal transition (EMT) in which tumor cells alter their morphology and behavior to become a much more invasive mesenchymal phenotype [13]. EMT has been shown to be a critical step in the dissemination of cancer cells [14] and the resulting mesenchymal like cells also express many characteristics of CSCs [12]. These two characteristics of increased drug resistance and increased invasion present a major challenge against the long term treatment and survivability of patients.

Cancer metastasis involves tumor cell invasion through a dense extracellular matrix. Invasion through such a dense matrix requires multiple steps for efficient migration, including translocation of the nucleus through very tight spaces [15]. Nuclear translocation is a major limiting factor for efficient 3D migration [15], and we have previously shown that non-muscle myosin IIB couples the nuclear scaffolding structures to the actomyosin cytoskeleton to facilitate squeezing the nucleus through tight spaces, allowing efficient 3D collagen invasion [16].

While previous work has been done to demonstrate that breast CSCs are more invasive than non-SCCs in the same cell line [7], there is very little evidence as to the mechanism that contributes to this increased invasion. For the current study, we utilized a NANOG-GFP reporter system in which we were able to compare migration properties of CSCs and non-SCCs that were isolated via flow cytometry from the same population of MDA-MB 231 cells. The previously described NANOG-GFP reporter can be used to isolate cells with elevated NANOG promoter activity. It was previously demonstrated that cells isolated via flow cytometry as having elevated promoter activity display elevated expression of endogenous NANOG, OCT4, and SOX2, and display CSC behavior in culture and in vivo [7]. Use of this reporter allows for comparison between genetically identical populations of cells to better identify aspects of invasiveness specific to the CSC population. We show here that upregulation of myosin IIB, concomitant with increase capacity to squeeze the nucleus through tight spaces, is a feature of the CSC subpopulation of MDA-MB 231 cells. This work provides possible new targets for investigations into therapeutic intervention in CSC-mediated pathology.

## RESULTS AND DISCUSSION

**Figure 1:**
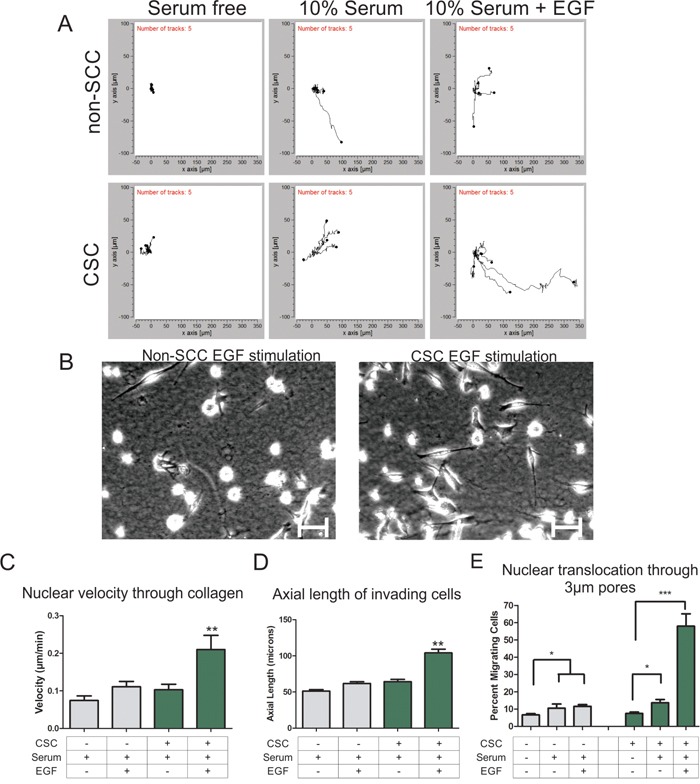
Cancer stem cells are more invasive through 3D collagen gel than non-stem cancer cells **A.** Individual migration tracks of nuclear positions as cells migrate through collagen towards the serum or EGF gradient (gradient source is to the right). **B.** Representative images of non-cancer stem cells (left) and cancer stem cells (right) as they migrate through collagen under EGF stimulation conditions. (Scale bars = 25 μm). **C.** Cell migration through 3D collagen gels was determined by tracking fluorescently labeled nuclei and the velocity was determined. CSCs are indicated with a “+” and non-SCCs are indicated with a “-“. **D.** The length of the longest protrusion was measured to determine the axial length of a cell as it migrated through the collagen gel. **E.** The percent nuclear translocation through 3 micron sized pores of a transwell membrane was determined by comparing the number of nuclei that crossed the membrane to the total number of cells plated. (* = P < 0.05, ** = P < 0.01, *** = P < 0.001)

Given earlier work from our lab and others that nuclear translocation is a critical rate-limiting step in 3D invasive migration in dense collagen [15–17], we asked if CSC populations differed from non-SCC populations in invasive ability. Three-dimensional collagen chemotaxis assays with CSC and non-SCC subpopulations reveal a similar modest stimulation of invasive migration in both subpopulation when stimulated with a 10% fetal bovine serum gradient (Figure 1A, left and center panels). To stimulate and direct invasion, a chemoattractant gradient of epidermal growth factor (EGF) was generated, which has been shown to be a potent inducer of invasion [18]. When an EGF gradient was applied, the CSC population displayed dramatically enhanced invasive migration that was not observed in the non-SCC population (Figure 1A, right panels, and Figure 1C). Analysis of the morphology of the cells invading through collagen revealed that the EGF stimulated CSCs produce longer protrusions than non-SCCs, leading to significantly longer and more extended cell morphology (Figure 1B and 1D). As a further measure of invasive behavior and competence for nuclear translocation, we analyzed the ability of cells to migrate through a transwell pores in response to a gradient of serum or EGF. A modest but significant increase in percent of transmigrating cells was seen in non-SCCs and CSCs stimulated with serum alone compared to unstimulated cells. While there was no significant increase in transmigrating non-SCCs upon stimulation with EGF compared to serum stimulation, there was a dramatic increase in transmigrating CSCs upon EGF stimulation (Figure 1E). These data suggest that EGF stimulation specifically enhances the invasive characteristics of CSCs by increasing protrusiveness and possibly by increasing competence for nuclear translocation as well.

**Figure 2:**
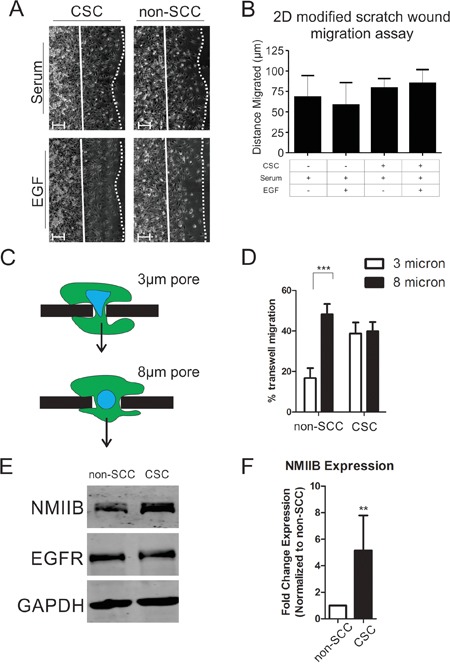
Cancer stem cells have a higher rate of invasion due to their enhanced ability for nuclear translocation **A.** Two-dimensional modified scratch wound assay representative images and **B.** quantification of distance migrated in 20 hours. Migration distance is quantified by measuring the distance from the starting position (solid lines) to the leading edge (dotted lines). Distances were averaged over 5 fields each of three independent replicates (n = 15; scale bars = 30 μm). CSCs are indicated with a “+” and non-SCCs are indicated with a “-“. **C.** Schematic of cellular migration through 3 vs. 8 micron size pores. Arrow indicates direction of migration. **D.** Nuclear translocation was assayed using 3 micron (white bars) or 8 micron (black bars) transwell membrane devices (*** = P < 0.001). All cells were stimulated with serum and EGF for 20 hours. **E.** Western blot comparing NMIIB and EGFR expression levels in non-SCCs to CSCs. **F.** Quantification of NMIIB expression via densitometric analysis of western blots relative to non-SCC. Values were normalized to GAPDH expression over five independent replicates and statistical analysis performed with Mann-Whitney analysis (** = P < 0.01).

**Figure 3:**
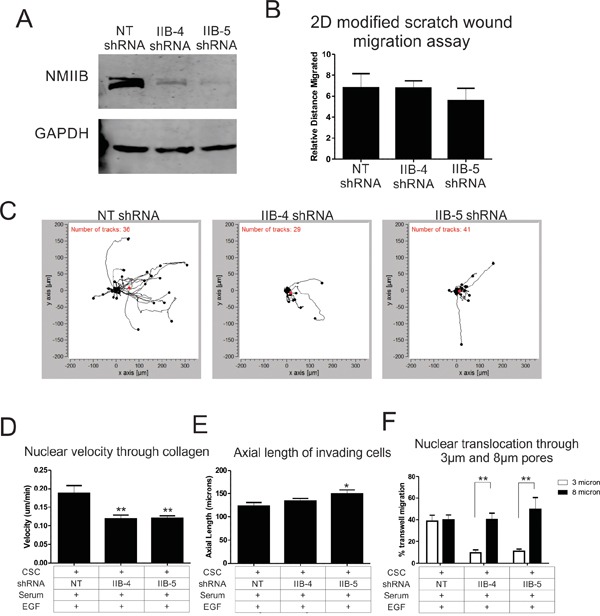
NMIIB is critical for enhanced motility of cancer stem cells **A.** Western blot of MDA-MB 231 cells infected with the NANOG-GFP reporter lentivirus was subsequently infected with lentivirus expressing a non-targeting (NT) control shRNA construct or two different NMIIB shRNA constructs. **B.** 2D migration rates were analyzed by PDMS peel comparing NT control shRNA to NMIIB shRNA cells. **C.** Individual cell tracks of CSCs were plotted for NT shRNA or NMIIB shRNA constructs as the cells invade through a collagen gel along an EGF gradient. EGF gradient source was to the right. **D.** The average nuclear velocity was quantified as cells invaded through collagen along an EGF gradient for 16 hours. CSCs are indicated with a “+” and non-SCCs are indicated with a “-“. **E.** The axial length of cells was quantified by measuring the length of the longest cellular protrusion at the 12 hour time point of collagen invasion. **F.** Nuclear translocation was analyzed by measuring the percent of nuclei that migrated through 3 micron (white bars) or 8 micron (black bars) size pores of a transwell membrane. (* = P < 0.05, ** = P < 0.01)

Increased invasiveness of CSCs could be due to an overall increase in motile behavior, or specifically due to upregulation of machinery related to 3D invasion. To determine if general motility behavior is upregulated in the CSC subpopulation, we analyzed the migration rates of CSCs versus non-SCCs in a previously described 2D modified scratch wound assay [19]. We observed no significant difference between CSC and non-SCC subpopulations with or without EGF stimulation (Figure 2A & 2B). To specifically test CSC and non-SCC populations for competence to translocate their nuclei through restrictive structures, we compared transwell migration through 3 μm pores (highly restrictive for nuclear translocation) versus less restrictive 8 μm pores [17, 20]. While both CSC and non-SCC cells were able to readily migrate through the 8 μm pores, the non-SCCs displayed significantly reduced ability to migrate through 3 μm pores (Figure 2C and 2D). The differential migratory ability of both CSCs and non-SCCs cannot be explained solely on EGFR expression levels, as both cell types express similar levels (Figure 2E). Based on our previous findings that NMIIB is critical for nuclear translocation [16], we hypothesized that increased invasiveness in the CSC population might be due to an upregulation of NMIIB levels. Western blot analysis (Figure 2E) and quantification by densitometry confirmed that NMIIB is significantly upregulated in the CSC population (Figure 2F). We therefore asked whether NMIIB is critical for the increased invasiveness of the CSC subpopulation. CSCs were infected with two different lentiviruses expressing NMIIB shRNA constructs (Figure 3A). Knockdown of NMIIB had no significant effect on 2D migration (Figure 3B), but it did significantly decrease overall invasive migration by CSCs in 3D collagen gels (Figure 3C & 3D). Interestingly, protrusiveness, as measured by axial length of NMIIB shRNA CSCs was not decreased and in fact displayed a modest yet significant increase compared to control (Figure 3E). This behavior is consistent with the model that NMIIB is critical for nuclear translocation in CSCs during invasive migration, but implies that protrusive responses to EGF are not impaired upon NMIIB shRNA. As a further test of nuclear translocation, we subjected to NMIIB shRNA cells to the comparative 3 and 8 micron pore size transwell assays. Consistent with the modified scratch wound migration results, CSCs were able to efficiently migrate through the non-restrictive 8 micron pores in response to an EGF gradient (Figure 3F, black bars). In contrast, NMIIB –depleted CSCs displayed a distinct impairment in transmigration through 3 micron pores (Figure 3F, white bars). We assessed this enhanced invasiveness of CSC populations in a different breast cancer cell line, HCC-70. Previous work demonstrated that introduction of the NANOG-GFP reporter into HCC-70 cells allowed identification of a CSC population [7]. In the current analysis, the CSC subpopulation of HCC-70 reporter cells demonstrated greater invasiveness through 3 μm transwell pores, greater transwell migration responsiveness to EGF, and elevated NMIIB expression (Supplementary Figure 1), confirming the behaviors observed in MDA MB-231 cells. Collectively, this analysis demonstrates a critical role for NMIIB in the enhanced invasive behavior of CSCs.

Only recently have studies begun to address the mechanics of tumor cell invasion in a physiological 3D environment. It is becoming clear that mechanisms exist for efficient migration through 3D extracellular matrices that are not realized when cells are studied in a 2D environment. Assays such as scratch wounds and wide bore transwell invasion chambers have yielded great details in cellular machinery and regulatory mechanisms, but do not present barriers for migration that a cell normally encounters as it invades through tissue. By analyzing mechanisms of cellular migration in more physiological settings, such as dense 3D collagen matrices and highly restrictive transwell pores can we more fully understand how cancer cells invade through the body and develop metastases. Cancer stem cells are therapeutic challenges that not only exhibit higher invasion to generate secondary metastases, but also generates metastases typically resistant to the therapies that were used to treat the primary tumor. The data presented in this work describe one mechanism in which CSCs enhance their ability to disseminate from the primary tumor. In order for efficient 3D invasion to occur, cancer stem cells exhibit increased levels of NMIIB which is critical for nuclear translocation through dense extracellular matrices. Further studies are needed to address other aspects of 3D invasion, such as machinery governing the increase in axial length and regulation of protrusions upon EGF stimulation of CSCs. Differential cytoskeletal activities of CSCs versus non-SCCs present potential therapeutic targets for inhibiting the dissemination of CSCs from the primary tumor, thereby preventing the generation of therapeutically resistant metastases.

## MATERIALS AND METHODS

### Cell culture

MDA-MB 231 cells were obtained from ATCC and all experiments were performed within 3 months of reconstitution of frozen stock. Cells were infected with pGreenZeo Nano-GFP Lenti-reporter construct (System Biosciences). To select for successful infection, cells were sorted for GFP expression using a FACS Aria-II cell sorting system. Sorted cells were then cultured for 2 weeks to allow for differentiation of non-SCCs. Cells were then sorted again into GFP positive and GFP negative populations, to enrich for CSCs and non-SCCs, respectively. To assure for pure populations, cells were only passaged for 2 weeks. MDA-MB 231 cells were cultured in Dulbecco's modified minimal essential media (DMEM) supplemented with 10% fetal bovine serum. Knockdown of NMIIB was achieved via lentiviral infection of shRNA constructs.

### Western blot analysis

Cells were trypsinized and washed in cold PBS before being lysed in 2X Laemmli sample buffer containing 0.5% beta-mercaptoethanol, 1% each of Protease Inhibitor Cocktail, Phosphatase Inhibitor Cocktail 2, and Phosphatase Inhibitor Cocktail 3 (Sigma-Aldrich, P8340, P5726, P0044, respectively). Samples were run on 4-20% Tris-Glycine gels (Life Technologies) and transferred to 0.45 μm PVDF-FL membrane (Millipore). Membranes were blocked in 2% milk solution and probed with the following antibodies: NMIIB (Biolegend, rabbit pAb; 1:1000 cat #909901), GAPDH (Santa Cruz Biotechnology rabbit pAb; 1:5000). EGFR (Cell Signaling Technology, rabbit pAb; 1:1000). Blots were analyzed with LiCor Odyssey CL imaging system.

### Collagen invasion assay

For tracking of cellular migration through collagen, cells were infected with pLV-RFP lentivirus expressing RFP-tagged histone 2B (Addgene #26001) to track nuclear movement. Following infection, CSCs and non-SCCs were isolated using flow based sorting, as described above. 3D collagen invasion assays were performed with Ibidi μ-slide Chemotaxis 3D chambers. Cells were embedded within 4 μg/ml collagen gel at 3×10^6^ cells/ml and loaded into the chamber. A chemoattractive gradient was then established with media containing either 10% serum or 10% serum plus 200 ng/ml EGF. Time lapse microscopy was performed on a Leica DMIRE 6000 inverted microscope at 37°C and 5% CO_2_ (10x/NA 0.7). Axial length was measured by tracing the length of the longest protrusion at the 12 hour time point in ImageJ. Velocity and cell tracks were measured in ImagePro Plus ver. 7. Tracks were automatically generated in ImagePro Plus by tracking movement of nuclei. Generated tracks were then analyzed using Ibidi Wimasis chemotaxis analysis software.

### Transwell invasion assay

3 and 8 micron pore size PET Transwell chambers (Becton Dickson) were placed in 24 well plates and coated with collagen. 5000 cells were then plated in the top reservoir of the chamber and allowed to adhere for 16 hours. Cells were then serum starved for 24 hours then stimulated by filling bottom reservoir with medium containing 10% serum or 10% serum plus 200 ng/ml EGF for 16 hours. Cells were then fixed and stained with DAPI. Total nuclei were imaged then cells in the upper portion of the membrane were then removed with a cotton swab. Membranes were then imaged again to quantify the percent of cells that migrated to the bottom side of the membrane.

### Modified scratch wound assay

Following methods previously described [19], 6 well plates were coated in collagen I (0.03 mg/ml). A thin strip of Polydimethylsiloxane (PDMS) was placed in the well. Cells were then plated in the well at a confluent level and allowed to adhere over-night. Cells were then serum starved for 20 hours and images of the cells with the PDMS peel in place were then gathered to determine starting positions. Cells were then stimulated with serum or serum plus EGF and the PDMS peel carefully removed. Images were then taken 20 hours post PDMS peel and distances calculated in ImageJ.

## SUPPLEMENTARY FIGURE


