# Supplementary material for: Increased cancer stem cell invasion is mediated by myosin IIB and nuclear translocation
Source: Oncotarget. 2016 Jun 7;7(30):47586–92. doi: 10.18632/oncotarget.9896 (PMC5216963; doi:10.18632/oncotarget.9896)
Supplement: Supplementary file 1 [file oncotarget-07-47586-s001.pdf]

Increased cancer stem cell invasion is mediated by myosin IIB and nuclear translocation

SUPPLEMENTARY FIGURE

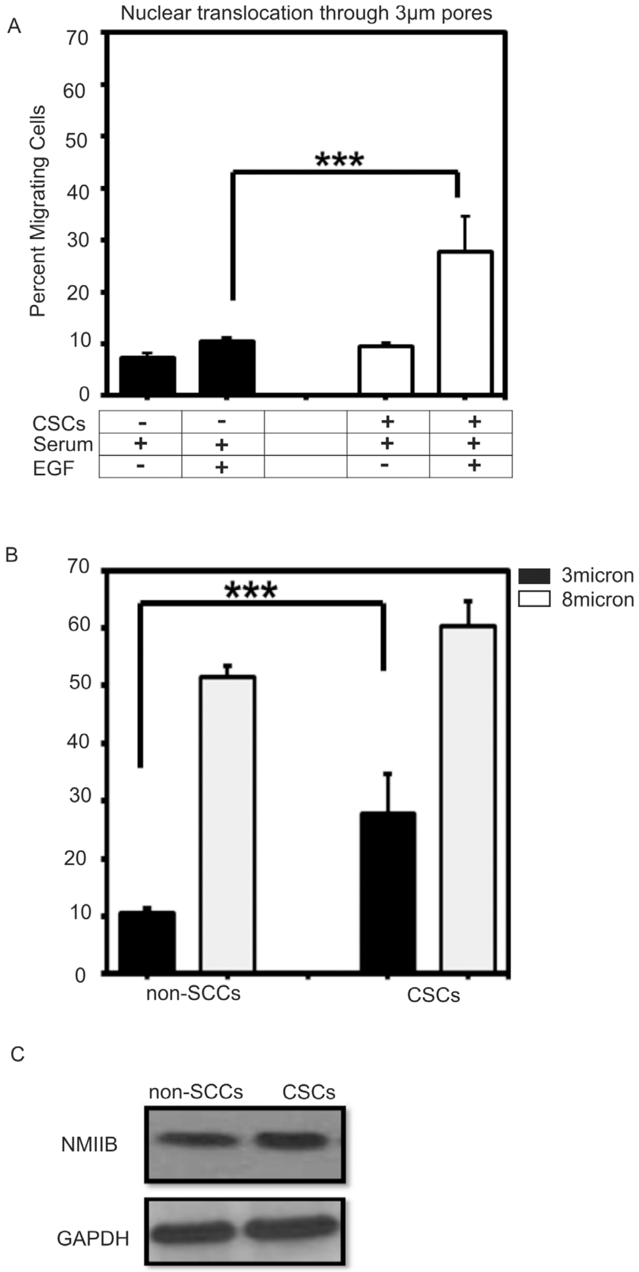

Supplementary Figure 1: CSCs derived from the HCC-70 breast cancer cell line display greater invasiveness and elevated NMIIB expression. HCC-70 cells were infected with the lentiviral construct carrying the NANOG-GFP reported, and sorted via flow cytometry as described [7]. **A.** The CSC and non-SCC subpopulations were evaluated for transwell migration through 3 μm pores +/- EGF stimulation. **B.** In the presence of EGF, migration of both CSC and non-SCC populations was similar through 8 μm pores (white bars), while CSCs displayed a statistically significant elevation in invasiveness through 3 μm pores. For (A) and (B) data were compiled from three independent replicates; $n = 15$ fields analyzed per condition. Error bars indicate SEM (***, $P < 0.001$) **C.** NMIIB is expressed at elevated levels in the CSC subpopulation of HCC-70 cells.
